# Microbial Diversity, Structure and Predicted Carbon-cycling Pathways Show Partial Convergence in Restored and Natural Salt Marshes in Venice Lagoon

**DOI:** 10.1007/s00248-026-02836-w

**Published:** 2026-07-15

**Authors:** Anjali Gopakumar, Md Masum Billah, Alessandro Vezzi, Fabio De Pascale, Davide De Battisti, Marco Bonato, Katherine A. Dafforn, Laura Airoldi

**Affiliations:** 1 Department of Biological, Geological, and Environmental Sciences, University of Bologna, Ravenna, 48123 Italy; 2 School of Natural Sciences, Macquarie University, Sydney, NSW 2109 Australia; 3 Inter-Departmental Research Centre for Environmental Science-CIRSA, University of Bologna, Ravenna Campus, Via S. Alberto 163, Ravenna, 48123 Italy; 4 Faculty of Fisheries, Atatürk University, 25240 Erzurum, Türkiye; 5 Department of Biology, University of Padova, Padova, 35121 Italy; 6 School for the Environment, University of Massachusetts Boston, Boston, MA 02125 USA; 7 NBFC, National Biodiversity Future Center, Palermo, 90133 Italy

**Keywords:** Salt marsh, Restoration, Sediment microbes, Predicted carbon cycling pathways, Mediterranean Sea

## Abstract

Salt marshes are increasingly restored following widespread degradation, yet the responses of sediment microbial communities to restoration remain poorly understood, despite their central role in ecosystem functioning. We compared microbial diversity, composition, structure and predicted function between natural (N) and two types of restored salt marshes in Venice lagoon, Italy: marshes restored with tidal creeks (RC), favouring a more natural tidal exchange and creek formation at the low marsh, and marshes restored with barriers (RB), which constrain tidal flow. We analysed sediment microbial communities in the three dominant habitat types of each marsh (unvegetated low marsh, vegetated low marsh, vegetated mid marsh) by amplicon sequencing of the V4 and V5 hypervariable regions of the 16 S rRNA gene. Microbial diversity was similar across natural and restored salt marshes, but community composition and structure were different. Natural marshes were characterised by higher abundances of taxa associated with sulphate reduction and organic matter degradation (e.g. *Syntrophobacterales*,* Bacteroidales* and *Desulfatiglandales*), whereas restored marshes showed greater representation of *Desulfobacterales*,* Desulfobulbales* and *Chromatiales*. In vegetated habitats, microbial communities also differed between low- and mid-marsh elevations, with low marshes enriched in *Syntrophobacterales* and *Desulfobulbales* and mid marshes characterised by *Ignavibacteriales* and *Defluviicoccales*. Despite these taxonomic differences, predicted carbon-cycling pathways were remarkably similar across restoration types and elevations. This suggests that key microbial functions can recover even when community composition remains distinct, indicating rapid re-establishment of microbial functional potential following restoration.

**Graphical Abstract:**

**Figure:**
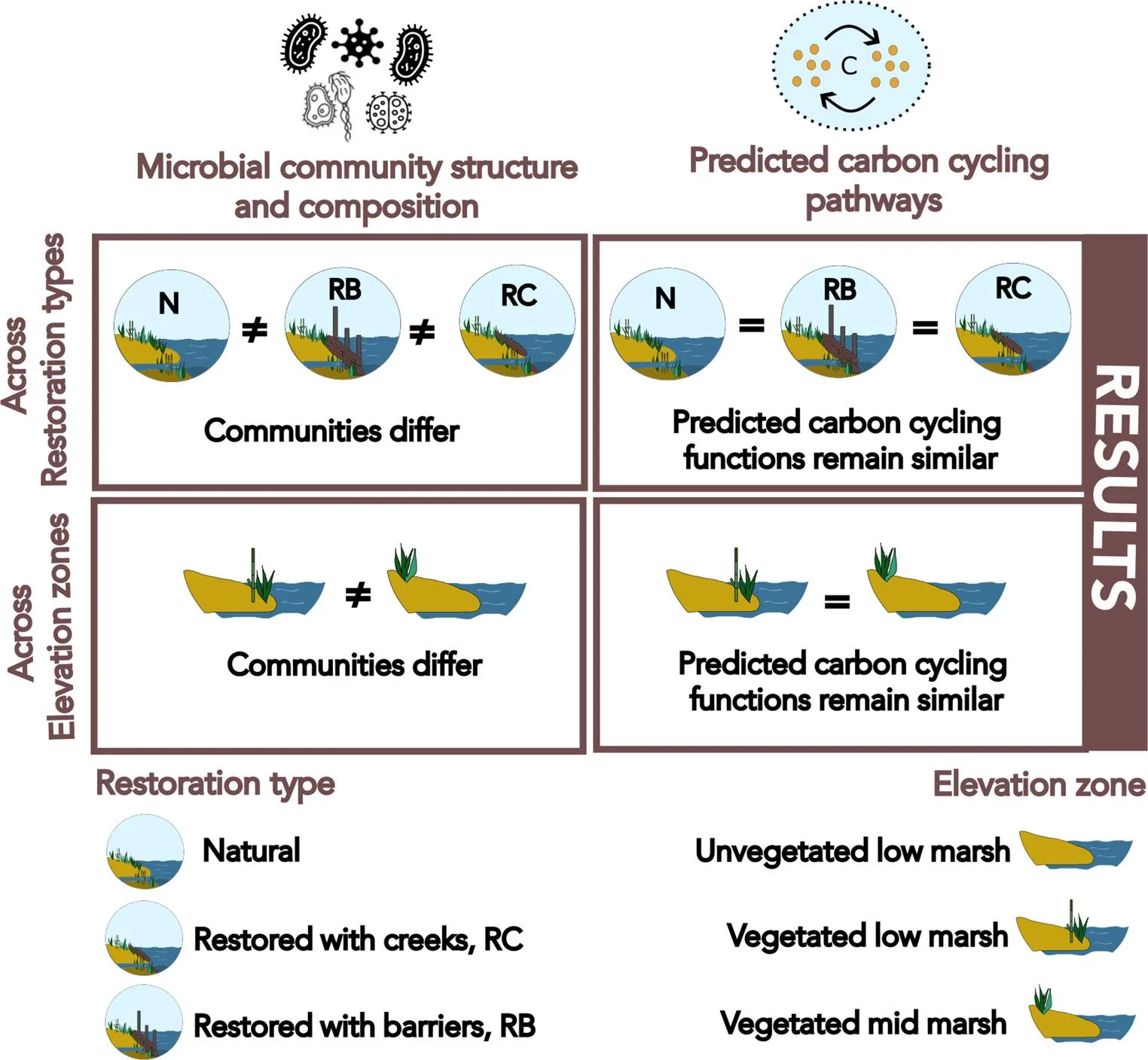

**Supplementary Information:**

The online version contains supplementary material available at https://doi.org/10.1007/s00248-026-02836-w.

## Introduction

Salt marshes are among the most productive coastal ecosystems in the world and provide vital ecosystem functions and services, including carbon sequestration, nutrient cycling, coastal protection, and water purification [1]. Over the centuries, these ecosystems have been strongly impacted by human activities, such as land reclamation and erosion, along with climate driven sea-level rise [2]. This has led to a rise in the number of restoration initiatives, including, for example, tidal restoration approaches via installation of tidal gates [3], the use of edge protection measures to prevent erosion [4], and de-embankment of seawalls to recreate salt marshes [5]. Studies on post-restoration efforts have predominantly focused on assessing the recovery of macro-scale structural and functional attributes such as halophytic vegetation cover and composition, sediment dynamics and productivity [6–8]. Few studies have explicitly tested whether different restoration designs lead to convergence in key micro-scale attributes such as the sediment microbiome and its functional potential [9].

Sediment microbial communities are critical to the functioning of salt marsh ecosystems, because they regulate biogeochemical cycling, organic matter, and carbon sequestration [10]. These communities are influenced by abiotic and biotic factors such as salinity, sediment type, elevation, nutrient concentrations, organic matter, and vegetation [11–13]. Elevation in particular plays a crucial role by determining the inundation frequency and thus influencing soil geochemistry and vegetation composition [10, 14], which in turn can alter the composition and diversity of sediment microbial communities [15].

Microbes can be grouped into different functional groups according to their predicted roles within ecosystems (e.g., methanogens, decomposers, etc.), as inferred from metabolic pathway analyses [16]. Many microbial populations can perform overlapping functions, a concept known as ‘functional redundancy’, which helps to maintain ecosystem processes even when some taxa are lost [17]. The loss of redundancy can reduce the resilience of the system and increase vulnerability to a variety of stressors, potentially disrupting microbial contributions to ecosystem functioning. For this reason, microbial communities are widely considered sensitive bioindicators of ecosystem processes and disturbances Changes in microbial diversity and community composition may therefore influence ecosystem functioning, making microbial communities useful indicators of environmental change and disturbance [18]. Hence, understanding the link between microbial community structure and ecosystem processes is essential for assessing resilience and sustaining ecosystem functioning [19].

Studies on microbiome responses to salt marsh restoration show inconsistent outcomes. Some studies report differences between restored and reference sites [20, 21], while others find similarities [17, 22]. Such inconsistencies may reflect environmental differences between restored and natural sites [6]. Microbial communities are highly sensitive to factors such as tidal regime and vegetation types, conditions which are strongly shaped by the restoration methods used. For example, many salt marshes are restored using dredged sediment and initially stabilised with protective barriers, which can change the elevation and alter inundation patterns, drainage, and early vegetation establishment. These physical alterations in turn influence sediment salinity, oxygenation, and grain size, thereby shaping the sediment microbiome. However, such conditions are difficult to replicate through restoration [8] and differences between restored and natural sites can persist for long periods [6]. Hence, understanding how restoration design and elevation, which are key drivers of inundation and sediment dynamics, shape microbial recovery is crucial for effective salt marsh management and restoration.

This study assessed whether alternative restoration designs lead to convergence of sediment microbial communities and their functional potential towards natural marsh conditions in the Venice lagoon (Italy), where extensive salt marsh restoration has been undertaken since 1985 [23, 24]. Building on previous evaluations of structural, functional and socio-economical restoration outcomes [4, 6, 23], we tested whether microbial diversity, community composition and predicted carbon-cycling functions recover similarly across restoration approaches.

We analysed the sediment microbial community diversity, structure, composition and predicted carbon cycling functions across replicated natural and restored marshes that had been established for over a decade. We compared two restoration methods: marshes restored with tidal creeks (RC), which promotes mudflat formation at the salt marsh edge, and marshes restored with barriers (RB), where intact barriers constrain tidal exchange and maintain steeper edges. Since natural marshes also lack engineered barriers, we hypothesized that RC salt marshes would support microbial communities more similar to those in natural (N) salt marshes than RB marshes [25], reflecting a more natural evolution of sediment characteristics at RC sites. Airoldi et al. [6] found that sediment carbon stocks were similar between natural (N) and restored (RC and RB) salt marshes, likely reflecting the carbon content of the dredged sediments rather than atmospheric carbon sequestration or the deposition of particulate organic matter during tidal inundation. Therefore, we expected the predicted carbon cycling pathways to converge functionally across the restoration types as well. We further explored differences between low and mid marsh habitats in RC and N marshes. We expected restored marshes to exhibit greater homogeneity in microbial communities across elevations, reflecting the more uniform sediment characteristics created by dredged sediments.

## Methods

### Study Area

**Fig. 1:**
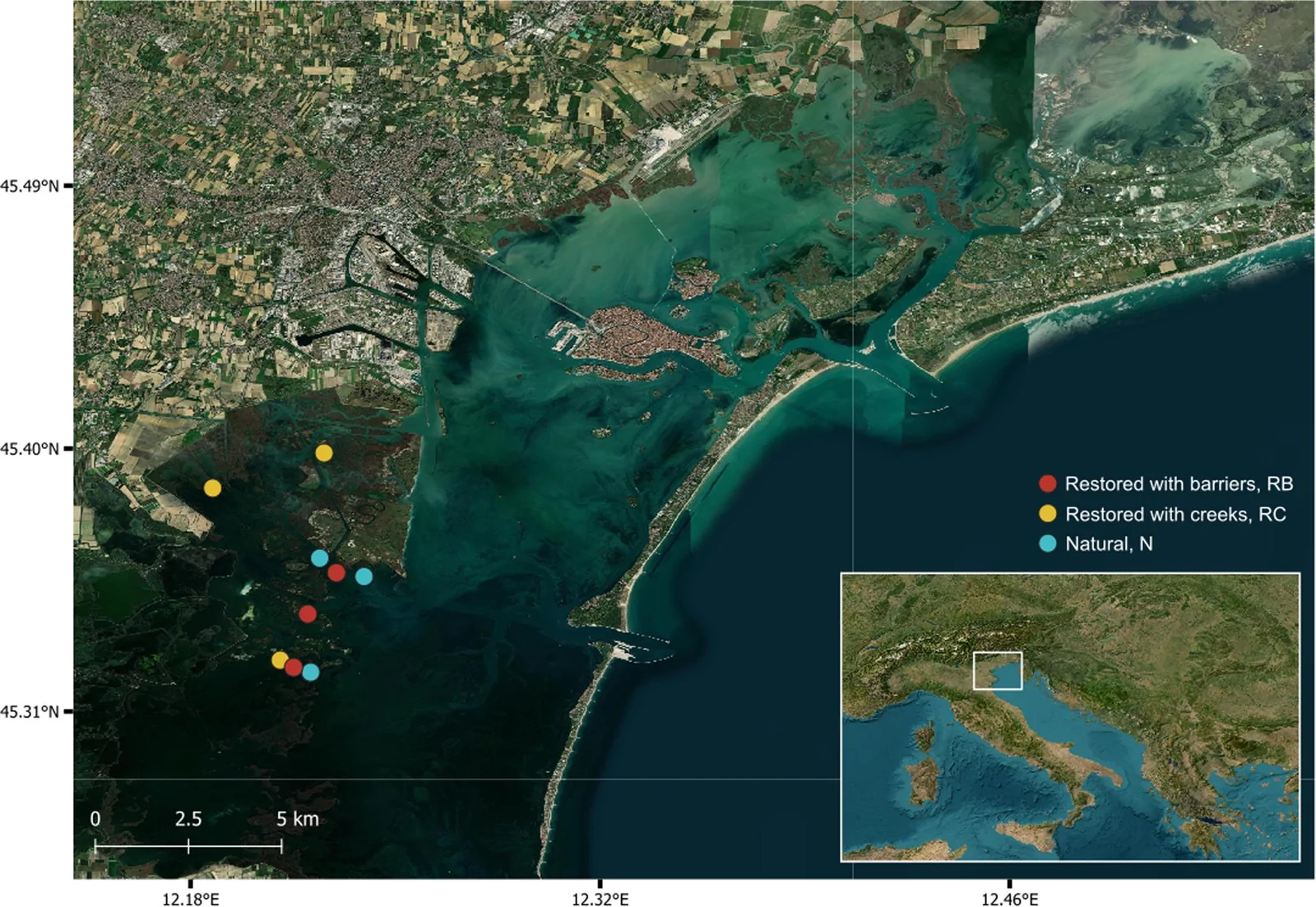
Natural and restored salt marshes sampled in the central basin of Venice lagoon, Italy (created using QGIS with ESRI World Imagery as a basemap; Source: Esri, Maxar, Earthstar Geographics, and the GIS User Community)

The study was conducted in the central Venice lagoon (Fig. 1: 45.3368° N, 12.2750° E), along the North-eastern coast of Italy. Human activities in and around the lagoon have led to a drastic loss and degradation of all habitats, including salt marshes, which were reduced by more than 70% between 1811 and 2002 [24]. To counteract these losses, efforts have been made to restore degraded salt marshes employing various approaches, including sediment pumping and containment barriers (like log poles, gabions or both) for edge protection. A detailed description of the study area is reported in [6].

### Sampling and Laboratory Analyses

We sampled three types of salt marshes within a section of the central Venice lagoon: Natural (N) marshes without visible containment structures, Restored with creeks (RC) salt marshes with partial edge protection measures alternating to naturally evolving low marsh with tidal creeks, and Restored with barriers (RB) salt marshes with full edge protection measures and abrupt edges. While these salt marshes are spatially separated, they occur within the same hydro-geomorphological setting and experience similar tidal regimes. For each type, three marshes over 10 years old and with similar sizes, dimensions, and hydrological conditions were selected over an area of approximately 3 × 10 km from this area, thus limiting large-scale spatial heterogeneity (Fig. 1, see details in [6]).

At each replicated marsh (hereafter Sites) microbial communities were sampled during low tide in May-June 2021 at two marsh elevations: mid marsh (> 30 cm above mean low water level) and low marsh (< 30 cm). The mid marsh was dominated by a mixed community of perennial species, including *Limonium narbonense*, *Puccinellia festuciformis*, and *Salicornia fruticosa* (hereafter ‘vegetated mid marsh’). At the low marsh, we sampled both unvegetated mudflats (‘unvegetated low marsh’) and patches of the largely established cordgrass *Spartina anglica* (Hubb) (or *Sporobolus anglicus*) [26] (‘vegetated low marsh’), which has largely replaced the native *Spartina maritima* in the region [27]. In RB marshes, however, the vegetated low marsh was not present [6], therefore sampling was naturally limited to unvegetated habitats. Because unvegetated habitats were also rare in the mid marsh of all marshes, a fully orthogonal comparison of vegetated and unvegetated habitats was not possible, reflecting the inherent structure of these restored systems. Therefore, two separate types of analyses were conducted: (a) differences among N, RC, and RB were examined in low marsh unvegetated habitats, and (b) differences between elevation zones and restoration effects were examined in vegetated habitats at N and RC.

In each habitat we randomly deployed four 50 × 50 cm quadrats for microbial community analysis. From each quadrat, we collected 3 sediment cores (diameter = 3 cm, depth 3 cm) from randomly selected corners using sterile, modified syringes (needle hub removed), and pooled the three cores into a 50 ml falcon tube. This pooled technique was used to obtain a composite sample representing the average microbial community within each quadrat, reducing the influence of small-scale spatial heterogeneity [28]. Fresh, sterile modified syringes were used at each replicate site to prevent contamination. In each quadrat, three additional cores were sampled using the same type of syringes for Chlorophyll-a analysis, and three large cores were sampled using a capped PVC corer (inner diameter = 9 cm, height = 20 cm) for organic matter, nitrogen and sulphur content, grain size, conductivity, and pH analyses. The methods and outcomes of these analyses have been described in detail in [6], where these data were used in a different context to compare the effectiveness of restoration at structural and macro-functional levels. Sediment for microbial analysis was sampled at a 3 cm depth to target surface sediment microbial communities, which are directly influenced by tidal inundation and other environmental changes, while sediment for physicochemical characteristics was sampled at 20 cm depth to represent broader, bulk sediment conditions. The samples were stored in portable freezers during their transport back to the laboratory at the nearby Chioggia hydrobiological station (45.2232° N, 12.2843° E). In the laboratory, each sediment sample intended for microbial analysis was homogenised, and an aliquot was transferred to a 2 mL Eppendorf tube and stored at − 80 °C prior to DNA extraction.

DNA was extracted from 0.25 g of each sediment sample using the DNeasy PowerSoil Pro kit (QIAGEN, Carlsbad, CA, USA), following the manufacturer’s guidelines, and DNA concentrations were quantified using a NanoDrop™ 2000 spectrophotometer (Thermo Fisher Scientific, Waltham, MA, USA). Amplification of the V4 and V5 hypervariable regions of the 16S rRNA gene was performed by polymerase chain reaction (PCR) using the specific primer pair 515F-Y (5’-GTGYCAGCMGCCGCGGTAA-3’) and 926R (5’-CCGYCAATTYMTTTRAGTTT-3’) [29]. The PCR conditions were as follows: 98° C for 4 min, 25 cycles of 98° C for 20 s, 57° C for 30 s, 72° C for 30 s, followed by a final extension at 72° C for 5 min. PCR products were purified using Agencourt^®^ AMPure XP beads (Agencourt Bioscience Corp., Beverly, MA, USA) and then tagged using the Nextera XT DNA sample preparation kit (Illumina Inc., San Diego, CA, USA) with a dual indexing strategy. A subsequent PCR with 12 cycles was performed to attach the index sequences to the purified amplicons. Following every PCR reaction, the quality of the extracted DNA was examined using agarose gel (1.5%) electrophoresis and the DNA concentrations were quantified using a NanoDrop™ 2000 spectrophotometer (Thermo Fisher Scientific, Waltham, MA, USA) or a Qubit™ 2.0 fluorometer (Thermo Fisher Scientific, Waltham, MA, USA). All the DNA extraction, amplification and purification steps were performed at the Department of Biology, University of Padova (Italy). Sequencing was carried out on a MiSeq sequencing platform paired-end 2 × 300 format (Illumina, San Diego, CA, USA), following the manufacturer’s guidelines.

The Illumina-sequenced data was imported into R via Rstudio and bioinformatic analysis was performed with the DADA2 pipeline implemented in the *dada2* package [30]. Briefly, following the removal of the 16 S amplification primers from the sequences using the *cutadapt* tool, the reads were trimmed and filtered (at 240 bp for forward reads and 160 bp for reverse reads), denoised, and merged to generate amplicon sequence variants (ASVs). Taxonomic assignment was performed on the resulting ASVs using the SILVA SSU database v.138 [31]. Non-target sequences (mitochondria, chloroplast, and eukaryotes), as well as unclassified reads were removed. Finally, low-abundance ASVs (< 10 total counts) and ASVs lacking taxonomic assignment at the phylum level were excluded prior to analysis to reduce noise and improve taxonomic reliability. All the raw sequences and meta-data have been uploaded to NCBI Sequence Read Archive (SRA) under the BioProject accession number: PRJNA1310060.

### Data Analysis

Due to the absence of vegetated low marsh habitats and the rarity of unvegetated mid marsh habitats, a fully orthogonal elevation zone × restoration analysis was not possible. Therefore, we conducted two separate analyses: (a) comparing N, RC and RB at low marsh unvegetated habitats; and (b) examining differences between elevation zones and restoration effects in vegetated habitats at N and RC. As microbial communities are strongly influenced by sediment characteristics, both comparisons were interpreted alongside the corresponding sediment data, which have been previously reported in [6].

The ASV table was normalised using Wrench [32], and the final ASV data, sediment characteristics and reference sequences were combined into a *phyloseq* object for further analysis, using the *phyloseq* package in R [33]. Alpha diversity indices (i.e., microbial diversity within a representative group) were calculated using the *phyloseq* package and only observed ASV richness and Shannon diversity were represented, because of a high correlation with and between the others (Simpson diversity and Chao1) (Fig. S1).

Generalised linear mixed-effect models using the *glmmTMB* function (from the *glmmTMB* package) [34] and a post-hoc *emmeans* tests [35] were used to compare the alpha diversity indices (ASV richness and Shannon diversity) in (a) unvegetated low marsh between restoration types (three levels: *N*,* RC and RB*) (no interaction with elevation zone) and (b) between restoration types (two levels: *N*,* RC*), elevation zone (two levels: *vegetated low marsh and mid marsh)*, and their interactions. In both analyses, Site was accounted for as a random factor nested in Restoration type. The normality of residuals and heterogeneity of variances were checked by plotting the residuals using the *DHARMa* package [36] in R, and transformations (log and inverse) were performed where applicable. An ANOVA (*car* package) [37] was performed on these models separately to obtain p-values.

The same logic was followed to analyse the multivariate differences in the microbial community structure and composition (microbial beta diversity, i.e., differences in microbial communities between groups) using the PERMANOVA function (*vegan* package) [38]. Bray-Curtis dissimilarity matrices were calculated from square-root transformed microbiome ASV data to analyse microbial community structure (based on ASV identity and abundance) and Jaccard dissimilarity matrices were calculated from presence-absence data to analyse microbial community composition (based on ASV identity only). Pairwise distinctions for analysis (a) were assessed using the *parirwise.adonis* function. *Betadisper* was run for both analyses to ensure the multivariate data fulfilled the criteria for homogenous dispersion. Additionally, the microbial taxa that characterised the microbial community structure in each group or that mostly contributed to the differences in significant PERMANOVA results (if any) were determined by a SIMilarity PERcentage analysis (SIMPER) (*vegan* package) [39]. Multivariate ordination for both analyses was performed using the unconstrained non-metric multidimensional scaling (nMDS) plot to visualise patterns in the microbial community structure, following which a constrained distance-based redundancy analysis (dbRDA) plot was constructed to visualise their relationships with relevant environmental variables (both plots built using Bray-Curtis dissimilarity matrices).

Similar to above, two separate analyses (for (a) and (b)) were run to predict differences in the functional potential of the microbial communities using the PICRUSt2 pipeline (Phylogenetic Investigation of Communities by Reconstruction of Unobserved States) (*picrust2_pipeline.py*) [40]. All ASVs were below the max NSTI cut-off of 2.0 and so all were retained for downstream analyses. Predicted functional pathways and their abundances were analysed using the enzyme commission numbering system, i.e., EC number and inferred from the MetaCyc Metabolic Pathway database [41]. These predicted pathways were filtered to keep pathways prevalent in > 10% of the samples and were further filtered to select only the pathways involved in carbon cycling. The abundances of these predicted pathways were CLR- transformed [42], and multivariate analyses were run by performing PERMANOVA (*vegan* package) [38] and assessing pairwise differences on Euclidean dissimilarity matrices. If PERMANOVA was significant, a multivariate *limma* test (*limma* package) [43] was run on the CLR-transformed predicted pathway composition data to identify which specific carbon-cycling pathways contributed to these community-level differences. nMDS plots were constructed for both analyses to visualise patterns in the functional pathways structure.

All data analyses were performed using R via Rstudio (version 4.3.0 [44]), except for PICRUSt2, that was run on the *conda* environment using Python. Plots were created using the *ggplot2* package in R [45]. Differences were considered significant at *p* < 0.05, except for SIMPER that focused on the most significant ASVs (*p* < 0.01).

## Results

### Differences among Restoration Types in Unvegetated Habitats

**Fig. 2:**
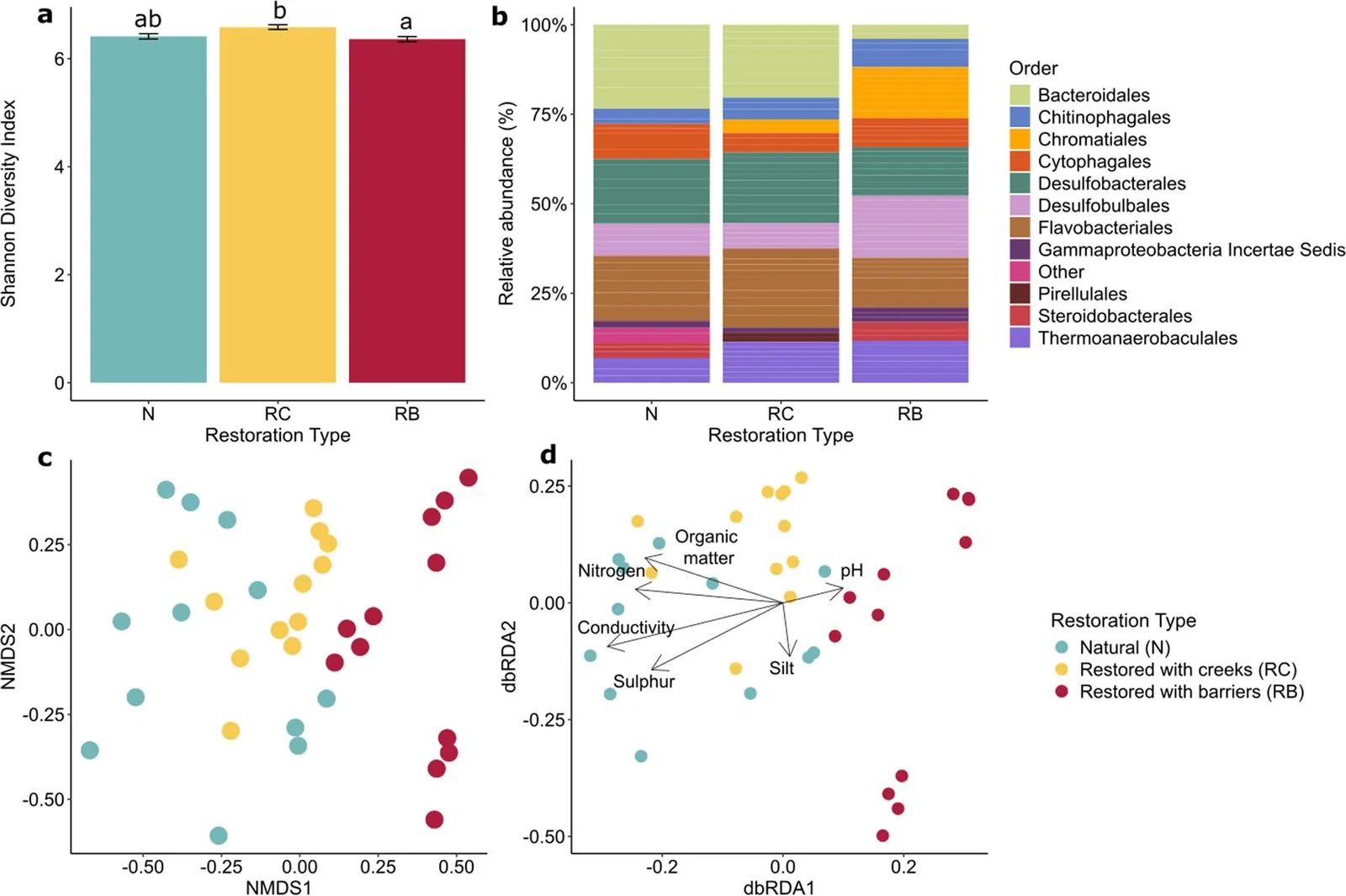
(**a**) Mean Shannon diversity measures in the unvegetated marsh habitat of natural (N), restored with creeks (RC) and restored with barriers (RB) salt marshes. The different lower-case letters above the bars indicate significant differences (*p* < 0.05) among salt marshes at each habitat type; (**b**) Relative abundances (%) of microbial orders for each restoration type. Microbial taxonomic orders with a relative abundance < 1% were grouped as ‘Other’; (**c**) Multivariate nMDS plot constructed using the Bray-Curtis dissimilarity index (Stress = 0.17) representing clusters of microbial community structure grouped by Restoration type (depicted by colour); and (**d**) distance-based redundancy analysis (dbRDA) plot, with fitted vectors representing sediment characteristics that explain the observed differences in microbial community structure

Microbial α-diversity was assessed by analysing the number of ASVs (i.e., observed microbial richness) and Shannon diversity in unvegetated low marsh habitats (Table S1). Sediment microbial richness did not differ between restoration types N, RC and RB ($\:{\chi\:}^{2}$=3.289, Df=2, *p*=0.193). Shannon diversity also did not differ between N and the two restored salt marshes, but it was higher in RC than RB (ChiSq = 9.900, DF=2, *p*< 0.001; Fig. 2a; Table S2). These patterns contrasted with those observed for the sediment characteristics, where most variables, including conductivity, organic matter, sulphur, silt-clay content, and pH differed between N, RC and RB (sediment details in [6]).

Microbial community structure and composition differed significantly between all restoration types, N, RC and RB (Fig. 2b and c; Table 1; Table S3). However, dispersion was heterogeneous between N, RC and RB for both structure and composition, indicative of variability within groups, hence results should be interpreted with caution. Differences in microbial community structure between N, RC and RB were best explained along the dbRDA1 axis, with higher nitrogen %, organic matter %, conductivity and total sulphur % associated with N marshes, low silt-clay concentration with RC and high pH associated with RB marshes (Fig. 2d; Table S4). SIMPER analysis showed that 13 bacterial ASVs (including *Syntrophobacterales*,* Bacteroidales*,* Desulfatiglandales*,* Desulfobacterales* and *Rhizobiales* orders) explained most of the differences between N and RC, 8 ASVs (linked to the orders *Syntrophobacterales*,* Desulfobulbales*,* Chromatiales* and *Run-SP154*) explained most of the differences between N and RB, and 5 ASVs (including *Chitinophagales*,* Chromatiales*,* Desulfobulbales*,* Run-SP154* and *Desulfobacterales*) explained most of the differences between RC and RB (Table 2). N salt marshes showed higher abundances of *Syntrophobacterales*,* Bacteroidales* and *Desulfatiglandales*, while RC had greater proportions of *Desulfobacterales*,* Rhizobiales* and *Desulfobulbales.* RB had higher proportions of *Desulfobulbales*,* Chromatiales*,* Desulfobacterales Run-SP154* and *Chitinophagales*, and was the only restoration type where *Pirellulales* was found. *Syntrophobacterales* contributed significantly to the dissimilarity between restoration types in the SIMPER analysis, despite its relatively low abundance and limited visibility in the stacked bar plot (Fig. 2b).

PICRUSt2 identified and annotated 510 predicted functional pathways in > 10% of the samples, out of which 78 were retained as predicted carbon cycling pathways. The overall predicted carbon cycling pathway composition differed significantly between N, RC and RB (Table 3; Fig. S2), with homogenous dispersions observed among groups. Pairwise PERMANOVA results indicated that these predicted pathways were different between N and RB (F = 3.416, R^2^ = 0.134, p-adj = 0.020), but not between the other restoration types (*p* > 0.05). However irrespective of these community-level differences, no individual predicted carbon cycling pathways were significantly different between the restoration types (Table S5).

**Table 1:** PERMANOVA and multivariate dispersion of (**a**) microbial community structure and (**b**) microbial community composition in response to restoration types (N: Natural, RC: Restored with creeks, RB: Restored with barriers) in unvegetated low marsh habitats. Significant effects (*p* < 0.05) are in bold

(a) Microbial community structure: PERMANOVA					
	**Df**	**SS**	**R** ^**2**^	**F**	**p**
Restoration type	2	1.132	0.199	7.438	**< 0.001**
Restoration type: Site	6	2.495	0.439	5.464	**< 0.001**
Residuals	27	2.055	0.362		
Permutation test for homogeneity of multivariate dispersion					
	**Df**	**SS**	**MS**	**F**	**p**
Groups	2	0.024	0.012	5.731	**0.01**
Residuals	33	0.068	0.002		
(b) Microbial community composition: PERMANOVA					
	**Df**	**SS**	**R** ^**2**^	**F**	**p**
Restoration type	2	1.393	0.167	5.002	**< 0.001**
Restoration type: Site	6	3.172	0.381	3.797	**< 0.001**
Residuals	27	3.759	0.452		
Permutation test for homogeneity of multivariate dispersion					
	**Df**	**SS**	**MS**	**F**	**p**
Groups	2	0.018	0.009	5.576	**0.007**
Residuals	33	0.052	0.002		

**Table 2:** Similarity percentage analysis (SIMPER) showing the ASVs contributing to the dissimilarities in microbial community structure between restoration types (N, RC, RB) in the unvegetated low marsh habitats. Average contribution indicates the contribution of the ASV to the total dissimilarity between groups. Av. of each group refers to the average abundances per group

(a) Between *N* and RC salt marshes					
ASV	Order	Average contribution	Standard deviation	Av. N	Av. RC
ASV2	*Syntrophobacterales*	0.0403	0.0312	0.015	0.008
ASV70	*Bacteroidales*	0.0324	0.0565	0.009	0.000
ASV164	*Desulfatiglandales*	0.0241	0.0396	0.007	0.001
ASV8	*Desulfobacterales*	0.0199	0.0124	0.005	0.007
ASV13	*Rhizobiales*	0.0156	0.0129	0.002	0.005
ASV102	*Flavobacteriales*	0.015	0.019	0.004	0.001
ASV156	*Bacteroidales*	0.013	0.013	0.003	0.002
ASV243	*Bacteroidales*	0.010	0.013	0.002	0.001
ASV138	*Chromatiales*	0.010	0.010	0.001	0.003
ASV586	*Desulfatiglandales*	0.010	0.012	0.003	0.000
ASV900	*Moduliflexales*	0.009	0.018	0.003	0.000
ASV12	*Steroidobacterales*	0.008	0.006	0.006	0.004
ASV245	*Flavobacteriales*	0.008	0.012	0.002	0.000
ASV1566	*Kiritimatiellales*	0.006	0.016	0.002	0.000
(b) Between N and RB marshes					
ASV	Order	Average contribution	Standard deviation	Av. N	Av. RB
ASV2	*Syntrophobacterales*	0.041	0.033	0.015	0.004
ASV5	*Desulfobulbales*	0.041	0.035	0.004	0.014
ASV33	*Chromatiales*	0.029	0.024	0.002	0.009
ASV16	*Desulfobulbales*	0.027	0.017	0.003	0.010
ASV154	*Run-SP154*	0.015	0.015	0.001	0.005
ASV66	*Chromatiales*	0.014	0.007	0.002	0.005
ASV279	*Desulfobacterales*	0.013	0.015	0.000	0.004
ASV20	*Thermoanaerobaculales*	0.013	0.009	0.004	0.006
(c) Between RC and RB marshes					
ASV	Order	Average contribution	Standard deviation	Av. RC	Av. RB
ASV60	*Chitinophagales*	0.031	0.043	0.001	0.009
ASV33	*Chromatiales*	0.030	0.024	0.002	0.009
ASV16	*Desulfobulbales*	0.023	0.016	0.003	0.010
ASV154	*Run-SP154*	0.015	0.016	0.001	0.005
ASV279	*Desulfobacterales*	0.013	0.015	0.001	0.004

**Table 3:** PERMANOVA and multivariate dispersion of the predicted carbon cycling pathway abundances across restoration types (N: Natural, RC: Restored with creeks, RB: Restored with barriers) in unvegetated low marsh habitats. Significant effects (*p* < 0.05) are in bold

	**Df**	**SS**	**R** ^**2**^	**F**	**p**
Model	8	694.26	0.526	3.750	**0.001**
Residual	27	624.79	0.474		
Permutation test for homogeneity of multivariate dispersion					
	**Df**	**SS**	**MS**	**F**	**p**
Groups	2	12.823	6.412	1.715	0.206
Residuals	33	123.356	3.738		

### Differences between Restoration Types and Elevation Zones in Vegetated Habitats

**Fig. 3:**
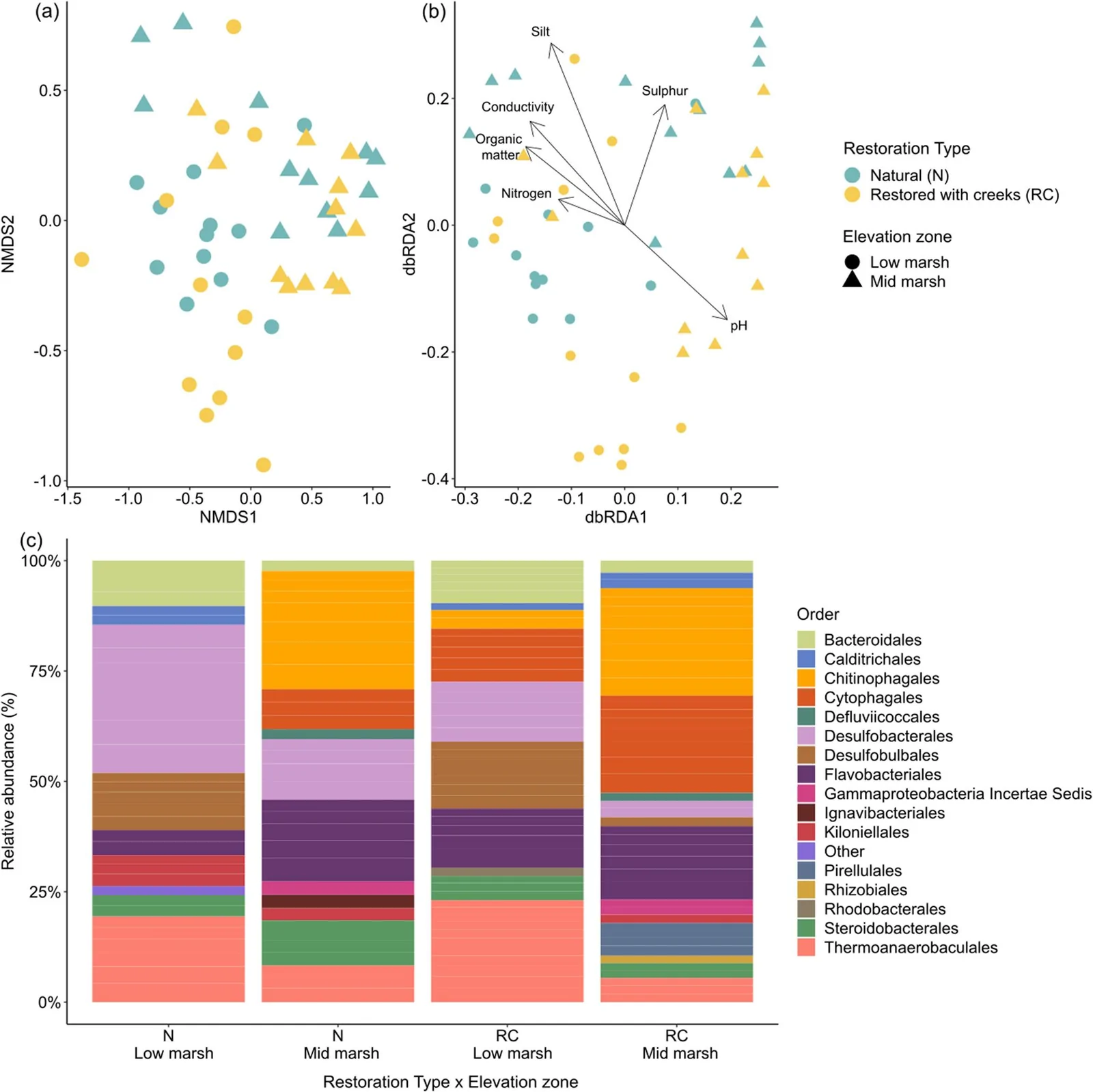
(**a**) Multivariate nMDS plot, based on Bray-Curtis dissimilarity (Stress = 0.11), representing clusters of microbial community structure grouped by Restoration type (depicted by colour: N: Natural, RC: Restored with creeks) and elevation zone (Low and Mid, depicted by shape); (**b**) distance-based redundancy analysis (dbRDA) plot, with fitted vectors representing sediment characteristics that explain the observed differences in microbial community structure; and (**c**) Relative abundances (%) of microbial orders for each combination of restoration type and elevation zone. Microbial orders with a relative abundance < 1% were grouped as ‘Other’

Microbial richness and Shannon diversity did not differ between restoration types (Natural, N, vs. Restored with creeks, RC) or elevation zones (vegetated low marsh vs. vegetated mid marsh) in vegetated marshes (Fig. S3; Table S6). In contrast, microbial community structure and composition consistently differed between N and RC (Fig. 3a and c; Table 4; Table S7) and between low and mid marsh (Fig. 3a and c; Table 4; Table S7), with no significant interaction between the two factors. Dispersion was homogeneous across groups (Table 4). Significant differences in microbial community structure between N and RC were best explained along the dbRDA1 axis, with higher nitrogen %, organic matter %, silt-clay content, conductivity, and chlorophyll concentration associated with N marshes, and higher pH and sulphur % associated with RC salt marshes (Fig. 3b; Table S8). Similarly, significant differences in microbial community structure between elevation zones were best explained along the dbRDA2 axis, with higher silt and organic matter % associated with mid marsh, and lower silt (i.e., finer sediments) and lower organic matter % associated with low marsh.

The distribution of key microbial taxa also differed between restoration types and elevation zones. Three bacterial ASVs (linked to the orders *Ignavibacteriales*, *Desulfobulbales* and *Desulfobacterales*) contributed most to the observed differences between N and RC (SIMPER analysis: Table 5a), with N marshes showing higher abundances of *Ignavibacteriales* and *Desulfobacterales*, and RC marshes showing higher abundances of *Desulfobulbales*. 41 ASVs accounted for the dissimilarities between elevation zones, with the 5 most influential ASVs belonging to the orders *Defluviicoccales*,* Syntrophobacterales*,* Desulfobulbales*,* Ignavibacteriales and Thermoanaerobaculales* (Fig. 3c; Table 5b). Vegetated low marshes showed higher abundances of *Syntrophobacterales*,* Desulfobulbales* and *Thermoanaerobaculales*, while vegetated mid marshes had greater proportions of *Ignavibacteriales* and *Defluviicoccales*. Predictive differential functional analysis (using PICRUSt2) found no statistically significant differences among restoration types (N, RC) across elevation zones (vegetated low marsh, vegetated mid marsh) (Table S9).

**Table 4:** PERMANOVA and multivariate dispersion of (**a**) microbial community structure and (**b**) microbial community composition in response to Restoration type (N: Natural, RC: with creeks) and Elevation zones (Low marsh vs. Mid marsh) in vegetated habitats. Significant effects (*p* < 0.05) are in bold

(a) Microbial community structure: PERMANOVA					
	**Df**	**SS**	**R** ^**2**^	**F**	**p**
Restoration type	1	0.536	0.0460	2.590	**< 0.001**
Elevation zone	1	1.687	0.145	8.154	**< 0.001**
Restoration type x Elevation zone	1	0.323	0.028	1.560	0.079
Residuals	44	9.106	0.781		
Permutation test for homogeneity of multivariate dispersion: Restoration type					
	**Df**	**SS**	**MS**	**F**	**p**
Groups	1	0.0001	0.0001	0.022	0.864
Residuals	46	0.212	0.005		
Permutation test for homogeneity of multivariate dispersion: Elevation zone					
	**Df**	**SS**	**MS**	**F**	**p**
Groups	1	0.0002	0.0002	0.018	0.901
Residuals	46	0.391	0.009		
(b) Microbial community composition: PERMANOVA					
	**Df**	**SS**	**R** ^**2**^	**F**	**p**
Restoration type	1	0.589	0.040	2.141	**< 0.001**
Elevation zone	1	1.578	0.108	5.735	**< 0.001**
Restoration type x Elevation zone	1	0.402	0.027	1.462	0.051
Residuals	44	12.107	0.823		
Permutation test for homogeneity of multivariate dispersion: Restoration type					
	**Df**	**SS**	**MS**	**F**	**p**
Groups	1	0.0009	0.0009	0.373	0.561
Residuals	46	0.114	0.002		
Permutation test for homogeneity of multivariate dispersion: Elevation zone					
	**Df**	**SS**	**MS**	**F**	**p**
Groups	1	0.0003	0.0003	0.073	0.785
Residuals	46	0.202	0.004		

**Table 5:** Similarity percentage analysis (SIMPER) showing the significant ASVs contributing to the dissimilarities in microbial community structure. Average contribution indicates the contribution of the ASV to the total dissimilarity between groups. Av. of each group refers to the average abundances per group

(a) Between *N* and RC salt marshes						
ASV	Order	Average contribution	Standard deviation	Av. N		Av. RC
ASV34	Ignavibacteriales	0.0248	0.0323	0.009		0.002
ASV40	Desulfobacterales	0.0122	0.0131	0.005		0.002
ASV5	Desulfobulbales	0.0122	0.0143	0.001		0.004
(b) Between low and mid marshes						
ASV	Order	Average contribution	Standard deviation	Av. mid marsh	Av. low marsh	
ASV4	*Defluviicoccales*	0.040	0.032	0.014	0.005	
ASV2	*Syntrophobacterales*	0.034	0.026	0.004	0.013	
ASV1	*Desulfobulbales*	0.029	0.026	0.002	0.010	
ASV34	*Ignavibacteriales*	0.026	0.034	0.009	0.001	
ASV3	*Thermoanaerobaculales*	0.019	0.014	0.005	0.009	
ASV21	*Cytophagales*	0.017	0.022	0.006	0.001	
ASV6	*Thermoanaerobaculales*	0.017	0.012	0.002	0.007	
ASV8	*Desulfobacterales*	0.016	0.013	0.002	0.006	
ASV62	*Desulfobacterales*	0.016	0.016	0.005	0.001	
ASV18	*Ignavibacteriales*	0.016	0.015	0.006	0.002	
ASV110	*Cytophagales*	0.015	0.020	0.005	0.000	
ASV10	*Thermoanaerobaculales*	0.013	0.009	0.002	0.006	
ASV54	*Gammaproteobacteria Incertae Sedis*	0.013	0.011	0.005	0.001	
ASV5	*Desulfobulbales*	0.013	0.014	0.001	0.004	
ASV17	*Calditrichales*	0.012	0.012	0.002	0.004	
ASV15	*Ectothiorhodospirales*	0.012	0.010	0.001	0.005	
ASV12	*Steroidobacterales*	0.012	0.007	0.002	0.005	
ASV13	*Rhizobiales*	0.012	0.010	0.002	0.005	
ASV136	*Thermoanaerobaculales*	0.012	0.012	0.004	0.000	
ASV22	*Thermoanaerobaculales*	0.012	0.012	0.001	0.004	
ASV7	*Cytophagales*	0.010	0.009	0.002	0.004	
ASV9	*Flavobacteriales*	0.010	0.009	0.002	0.004	
ASV202	*Thalassobaculales*	0.010	0.011	0.003	0.000	
ASV24	*Chitinophagales*	0.010	0.012	0.003	0.001	
ASV128	*BD7-8*	0.009	0.008	0.003	0.001	
ASV155	*Polyangiales*	0.009	0.008	0.003	0.001	
ASV162	*Pseudomonadales*	0.009	0.009	0.003	0.000	
ASV76	*Chitinophagales*	0.008	0.010	0.003	0.001	
ASV265	*Desulfobulbales*	0.008	0.010	0.002	0.000	
ASV31	*Cytophagales*	0.007	0.010	0.003	0.001	
ASV232	*Cytophagales*	0.007	0.013	0.002	0.000	
ASV95	*Cytophagales*	0.007	0.009	0.002	0.001	
ASV372	*Flavobacteriales*	0.005	0.007	0.002	0.000	
ASV495	*Gammaproteobacteria Incertae Sedis*	0.005	0.011	0.002	0.000	
ASV214	*Flavobacteriales*	0.005	0.009	0.002	0.000	
ASV584	*NA (Class: Gammaproteobacteria)*	0.005	0.009	0.002	0.000	
ASV206	*Cytophagales*	0.004	0.011	0.001	0.000	
ASV392	*Calditrichales*	0.004	0.014	0.001	0.000	
ASV226	*Cytophagales*	0.004	0.009	0.001	0.000	
ASV354	*Cytophagales*	0.003	0.008	0.001	0.000	
ASV501	*Calditrichales*	0.003	0.007	0.001	0.000	
ASV955	*Thermoanaerobaculales*	0.002	0.007	0.001	0.000	

## Discussion

In this study, sediment microbial communities were used to assess the outcomes of salt marsh restoration efforts in Venice lagoon. Previous work found that restoration methods allowing natural foreshore dynamics (RC) facilitated low-marsh vegetation establishment more effectively than barrier-based designs (RB), while mid‑marsh indicators, such as soil carbon and vegetation structure were comparable across restoration types [6]. Building on these findings, we show that although microbial community structure and composition differed among natural (N), RC and RB marshes, microbial richness, diversity and predicted carbon‑pathway were broadly similar, indicating functional convergence across restoration types.

Microbial recovery is critical for restoring salt marsh functions, and previous studies have shown that microbial communities in restored marshes can converge towards natural reference conditions within a few years [20, 46]. Consistently, we found similar microbial richness and diversity between restored and natural marshes in both unvegetated and vegetated habitats. In contrast, microbial community structure and composition differed across restoration types and elevation zones, reflecting persistent differences in sediment characteristics. N marshes were more saline, acidic, nutrient-rich, and finer grained than restored marshes [6], and these environmental differences likely shaped microbial assemblages.

We expected RC marshes to show reduced differences across elevation zones due to sediment homogeneity. Instead, both N and RC marshes exhibited clear structural microbial differences between mid and low marsh vegetated habitats. These differences were associated with higher silt content and organic matter at mid marsh and lower values at low marsh, reflecting potential hydrological and geochemical gradients. This suggests that environmental gradients can rapidly re-establish microbial zonation even after large-scale disturbance, supporting previous evidence of microbial resilience [47]. Sediment characteristics emerged as key drivers of microbial community structure, in line with studies showing that abiotic restoration outcomes indirectly shape microbial recovery [17].

Taxonomic differences between restoration types were driven mainly by groups involved in sulphur cycling and organic matter degradation, including sulphate-reducers and decomposers. Despite these compositional shifts, predicted carbon cycling pathways did not differ consistently among restoration types or across elevation zones. Moreover, although overall pathway abundances differed between some restoration types (notably N and RB), no individual pathway differed significantly, indicating convergence at the functional level. This pattern is consistent with previous observations of comparable sediment carbon stocks across restoration types [6].

Together, these results suggest a functional convergence across restoration types, where different taxa perform similar ecological roles despite persistent compositional differences driven by environmental conditions [17]. Fundamental microbial processes related to carbon turnover, such as decomposition, fermentation, methanogenesis, and carbon fixation, appear broadly similar in restored and natural marshes. While restoration success is increasingly evaluated in terms of ecosystem functions rather than taxonomic composition [9, 48], functional endpoints remain difficult to measure directly in microbial systems. Therefore, functional inferences based on taxonomic predictions should be interpreted cautiously. Nevertheless, the observed functional convergence provides encouraging evidence that restoration in the Venice Lagoon can recover key microbial ecosystem functions.

RC and RB showed microbial richness and diversity comparable to N marshes, although Shannon diversity was slightly higher in RC than RB marshes, likely reflecting the more natural tidal exchange. These patterns suggest that restoration interventions in the Venice lagoon are leading to marshes with microbial attributes characteristic of functionally established systems. This is particularly promising given that poorly designed restorations may fail to reach functional equivalence [48].

Finally, although several indicators converged across natural and restored salt marshes, including microbial diversity, predicted carbon pathways, soil carbon and vegetation structure [6], restoration designs that restrict hydrodynamics (like RB) may reduce long-term resilience. Designs that mimic natural tidal processes are therefore likely to support more robust recovery [4]. Improved understanding of sediment microbiome responses to restoration remains a priority [49], and long-term monitoring will be essential to assess successional trajectories and resilience under different restoration approaches.

Overall, we used amplicon sequencing to show that although sediment microbial communities remain taxonomically distinct across restoration types in Venice lagoon, their predicted functional profiles, particularly carbon-cycling pathways, have converged. Microbial richness and diversity were comparable between natural and restored salt marshes across both vegetated and unvegetated habitats, while community composition differed. Importantly, although different taxa contributed to these differences, their predicted functions were similar, indicating functional convergence across restoration types. These findings suggest that complete taxonomic recovery may not be necessary for functional restoration, as distinct microbial assemblages can fulfil similar ecological roles. This supports the paradigm that ecosystem function is a more relevant metric of restoration success than community composition alone [48, 50]. However, stronger inference of functional recovery will require direct measures of microbial activity. Future work should integrate functional assays and meta-transcriptomic approaches to link community composition with expressed metabolic functions and to better evaluate long-term ecosystem resilience.

## Supplementary Information

Below is the link to the electronic supplementary material.

Supplementary Material 1

## Data Availability

All the raw sequences and meta-data have been uploaded to NCBI Sequence Read Archive (SRA) under the BioProject accession number: PRJNA1310060. The datasets and codes generated during and/ or analysed during the current study are available in the GitHub repository named ‘Microbes-in-Venice-salt-marshes’, available at the link [https://github.com/anjalig95/Microbes-in-Venice-salt-marshes.git](https:/github.com/anjalig95/Microbes-in-Venice-salt-marshes.git) . The rest of the data generated or analysed during this study are included in this published article and its supplementary information files.
