# Supplementary material for: Microbial Diversity, Structure and Predicted Carbon-cycling Pathways Show Partial Convergence in Restored and Natural Salt Marshes in Venice Lagoon
Source: Microb Ecol. 2026 Jul 15;89:171. doi: 10.1007/s00248-026-02836-w (PMC13550280; doi:10.1007/s00248-026-02836-w)
Supplement: Supplementary file 1 [file 248_2026_2836_MOESM1_ESM.docx]

**SUPPLEMENTARY INFORMATION**

**Microbial diversity, structure and predicted carbon-cycling pathways show partial convergence in restored and natural salt marshes in Venice lagoon**

Anjali Gopakumar^1,2*^, Md Masum Billah^3,4^, Alessandro Vezzi^5^, Fabio De Pascale^5^, Davide De Battisti^5^, Marco Bonato^5^, Katherine A. Dafforn^2,6^, Laura Airoldi^5,7^

^1^Department of Biological, Geological, and Environmental Sciences, University of Bologna, Ravenna – 48123, Italy

^2^School of Natural Sciences, Macquarie University, Sydney, NSW – 2109, Australia  ^3^Inter-Departmental Research Centre for Environmental Science-CIRSA, University of Bologna, Ravenna Campus, Via S. Alberto 163, Ravenna – 48123, Italy.  ^4^Faculty of Fisheries, Atatürk University, Erzurum – 25240, Türkiye

^5^Department of Biology, University of Padova, Padova – 35121, Italy

^6^School for the Environment, University of Massachusetts Boston, Boston, MA – 02125, USA ^7^NBFC, National Biodiversity Future Center, Palermo – 90133, Italy

*Corresponding author: Dr. Anjali Gopakumar, 12 Wally’s Walk, Macquarie University, North Ryde, NSW – 2109, Australia; Email: [anjali.gopakumar@mq.edu.au](mailto:anjali.gopakumar@mq.edu.au); Phone: +61-452498757; ORCID ID: 0000-0001-5642-4951

**Fig. S1** Chart correlation of all alpha diversity indices measured in this study: Observed = Observed richness; Chao1 = Chao1 index; Shannon = Shannon diversity index; InvSimpson = Simpson’s diversity index.

**Fig. S2** (a) Multivariate nMDS plot, based on Euclidean dissimilarity representing clusters of CLR-transformed predicted carbon cycling pathway abundances grouped by Restoration type (depicted by colour: N: Natural, RC: Restored with creeks; RB: Restored with barriers) and Elevation zone (low marsh and mid marsh, depicted by shape) in (a) unvegetated low marshes (Stress = 0.16) and (b) vegetated low and mid marshes (Stress = 0.14).

**Fig. S3** Mean (a) Observed ASV richness and (b) Shannon diversity measures in the two restoration types (natural (N) and restored with creeks (RC)) and the two elevation zones (vegetated low marsh and vegetated mid marsh).

**Table S1** Microbial diversity measures, vegetation composition and physicochemical characteristics of sediment from each restoration type and elevation zone. The values are expressed as mean ± standard deviation.

| Restoration type | Elevation zone | Vegetation species | Observed richness | Shannon diversity | Conductivity (μS/cm) | pH | Nitrogen (%) | Sulphur (%) | Organic matter (%) | Silt-clay (%) | Chlorophyll-a (µg/g) |
| --- | --- | --- | --- | --- | --- | --- | --- | --- | --- | --- | --- |
| Natural (N) | Unvegetated low marsh | NA | 1112.583  ±304.442 | 6.413 ±0.173 | 10618.08  ±3632.78 | 7.91  ±0.23 | 0.27  ±0.12 | 0.94  ±0.31 | 15.03  ±2.61 | 80.1  ±20.1 | 13.83  ±8.04 |
| Natural (N) | Vegetated low marsh | *Sporobolus anglicus* | 1240.833 ±  184.781 | 6.613 ±0.162 | 12396.5  ±3458.52 | 7.81  ±0.12 | 0.32  ±0.09 | 0.62  ±0.36 | 16.92  ±2.71 | 74.47  ±30.52 | 14.66  ±5.77 |
| Natural (N) | Vegetated mid marsh | *Limonium narbonense; Puccinellia festuciformis; Salicornia fruticosa* | 1154 ±  164.582 | 6.582 ±0.171 | 19271.67  ±4835.09 | 7.66  ±0.26 | 0.53  ±0.12 | 0.77  ±0.38 | 23.58  ±2.58 | 85.06  ±14.33 | 32.01  ±10.26 |
| Restored with creeks (RC) | Unvegetated low marsh | NA | 435.25 ±  346.685 | 6.585 ±0.154 | 6170.36  ±2955.34 | 8.16  ±0.22 | 0.19  ±0.08 | 0.47  ±0.26 | 12.44  ±2.38 | 44.44  ±25.09 | 12.36  ±6.47 |
| Restored with creeks (RC) | Vegetated low marsh | *Sporobolus anglicus* | 1382.167 ±  159.853 | 6.686 ±0.132 | 5055.75  ±3409.53 | 8.36  ±0.34 | 0.28  ±0.17 | 0.34  ±0.28 | 14.27  ±3.07 | 37.95  ±32.08 | 14.13  ±9.3 |
| Restored with creeks (RC) | Vegetated mid marsh | *Limonium narbonense; Puccinellia festuciformis; Salicornia fruticosa* | 1336.417 ±  415.153 | 6.577 ±0.348 | 6143  ±4691.98 | 8.12  ±0.3 | 0.27  ±0.14 | 0.3  ±0.26 | 14.85  ±5.50 | 27.84  ±33.51 | 20.58  ±10.13 |
| Restored with barriers (RB) | Unvegetated low marsh | NA | 1112.833 ±  201.388 | 6.362 ±0.164 | 4140.5  ±2048.92 | 8.07  ±0.14 | 0.14  ±0.06 | 0.4  ±0.21 | 10.54  ±1.97 | 76.45  ±21.71 | 8.56  ±4.53 |

**Table S2** Pairwise comparisons (using Estimated Marginal Means) of microbial Shannon diversity in the unvegetated low marsh among the three restoration types (N: Natural, RC: Restored with creeks, RB: Restored with barriers). Significant effects (p <0.05) are in bold.

| Restoration type | Groups | Estimate | SE | Df | t | p-value |
| --- | --- | --- | --- | --- | --- | --- |
| Shannon diversity | | | | | | |
| Unvegetated low marsh | N – RC | -0.172 | 0.074 | 31 | -2.314 | 0.069 |
|  | N – RB | 0.051 | 0.074 | 31 | 0.689 | 0.772 |
|  | RC > RB | 0.223 | 0.074 | 31 | 3.003 | **0.014** |

**Table S3** Pairwise results of PERMANOVA comparing (a) microbial community structure and (b) microbial community composition between the restoration types (N: Natural, RC: Restored with creeks, RB: Restored with barriers). Significant effects (p <0.05) are in bold.

(a) **Microbial community structure**

| Pairwise distinctions between restoration types | | | | | | |
| --- | --- | --- | --- | --- | --- | --- |
| Pairs | **Df** | **SS** | **F** | **R^2^** | **p** | **p-adj** |
| N – RC | 1 | 0.322 | 2.297 | 0.095 | 0.005 | **0.005** |
| N – RB | 1 | 0.762 | 5.138 | 0.189 | <0.001 | **<0.001** |
| RC – RB | 1 | .614 | 4.906 | 0.182 | <0.001 | **<0.001** |

(b) **Microbial community composition**

| Pairwise distinctions between restoration types | | | | | | |
| --- | --- | --- | --- | --- | --- | --- |
| Pairs | **Df** | **SS** | **F** | **R^2^** | **p** | **p-adj** |
| N – RC | 1 | 0.438 | 2.061 | 0.086 | 0.002 | **0.002** |
| N – RB | 1 | 0.867 | 3.920 | 0.151 | <0.001 | **<0.001** |
| RC – RB | 1 | 0.784 | 3.991 | 0.154 | <0.001 | **<0.001** |

**Table S4** ANOVA output from the distance-based redundancy analysis (dbRDA) plot, showing the sediment characteristics that explain the differences observed in the microbial community structure in the between restoration types (N: Natural, RC: Restored with creeks, RB: Restored with barriers) in the unvegetated habitats. Significant effects (p < 0.05) are in bold.

|  | Df | SS | F | p |
| --- | --- | --- | --- | --- |
| Nitrogen | 1 | 0.564 | 4.638 | **0.001** |
| Conductivity | 1 | 0.210 | 3.330 | **0.001** |
| pH | 1 | 0.207 | 1.699 | 0.054 |
| Sulphur | 1 | 0.284 | 2.332 | **0.006** |
| Silt-clay content | 1 | 0.295 | 2.427 | **0.003** |
| Organic matter | 1 | 0.402 | 3.304 | **0.001** |
| Residuals | 29 | 3.526 |  |  |

**Table S5** Combined results of differential pathway analysis performed using limma on CLR‑transformed PICRUSt2‑predicted carbon‑cycling pathways, in the unvegetated low marsh habitats.

| Pairwise contrasts | Pathway ID | logFC | AveExpr | t | p | p-adj | B |
| --- | --- | --- | --- | --- | --- | --- | --- |
| N vs RC | PWY-3481 | 1.030 | -2.917 | 1.387 | 0.174 | 0.826 | -4.490 |
|  | FERMENTATION-PWY | -0.146 | 1.977 | -1.292 | 0.205 | 0.826 | -4.526 |
|  | P461-PWY | -0.272 | 0.105 | -1.274 | 0.211 | 0.826 | -4.533 |
|  | PWY-7111 | -0.140 | 2.423 | -1.266 | 0.214 | 0.826 | -4.535 |
|  | CALVIN-PWY | -0.144 | 2.501 | -1.229 | 0.227 | 0.826 | -4.549 |
|  | PWY-5209 | 0.448 | -0.701 | 1.203 | 0.237 | 0.826 | -4.558 |
|  | PWY-6435 | 0.167 | 1.008 | 1.164 | 0.253 | 0.826 | -4.571 |
|  | PWY-6641 | -0.281 | 1.226 | -1.163 | 0.253 | 0.826 | -4.571 |
|  | PWY-5742 | 0.228 | -0.852 | 1.140 | 0.262 | 0.826 | -4.579 |
|  | P23-PWY | -0.128 | 2.449 | -1.131 | 0.266 | 0.826 | -4.582 |
|  | P42-PWY | -0.146 | 2.557 | -1.125 | 0.268 | 0.826 | -4.584 |
|  | GLUCONEO-PWY | -0.131 | 2.401 | -1.096 | 0.281 | 0.826 | -4.593 |
|  | PWY-5384 | -0.125 | 2.350 | -1.090 | 0.283 | 0.826 | -4.595 |
|  | PWY-5130 | -0.157 | 2.141 | -1.067 | 0.293 | 0.826 | -4.603 |
|  | PWY-7431 | -0.187 | 0.444 | -1.064 | 0.295 | 0.826 | -4.603 |
|  | NONOXIPENT-PWY | -0.126 | 2.653 | -1.054 | 0.299 | 0.826 | -4.607 |
|  | PWY-6969 | -0.128 | 2.578 | -1.051 | 0.301 | 0.826 | -4.607 |
|  | PWY-8178 | -0.129 | 2.652 | -1.041 | 0.305 | 0.826 | -4.610 |
|  | PWY-6628 | -0.113 | 2.330 | -1.035 | 0.308 | 0.826 | -4.612 |
|  | COMPLETE-ARO-PWY | -0.113 | 2.473 | -1.033 | 0.309 | 0.826 | -4.613 |
|  | PWY66-373 | 0.318 | -2.172 | 1.015 | 0.317 | 0.826 | -4.618 |
|  | PWY-5659 | -0.110 | 2.531 | -0.974 | 0.337 | 0.826 | -4.630 |
|  | REDCITCYC | 0.203 | 1.132 | 0.957 | 0.345 | 0.826 | -4.635 |
|  | PWY0-321 | 0.136 | 0.367 | 0.951 | 0.348 | 0.826 | -4.637 |
|  | PWY-5430 | 0.188 | -0.670 | 0.949 | 0.349 | 0.826 | -4.637 |
|  | PWY-5022 | -0.154 | 1.239 | -0.943 | 0.352 | 0.826 | -4.639 |
|  | GLYCOLYSIS | -0.105 | 2.427 | -0.935 | 0.356 | 0.826 | -4.641 |
|  | P163-PWY | -0.231 | 0.064 | -0.912 | 0.368 | 0.826 | -4.647 |
|  | PWY-7323 | -0.108 | 2.244 | -0.907 | 0.371 | 0.826 | -4.649 |
|  | PWY-8187 | -0.163 | 1.586 | -0.902 | 0.373 | 0.826 | -4.650 |
|  | PWY-5484 | -0.097 | 2.360 | -0.886 | 0.382 | 0.826 | -4.655 |
|  | PWY-1042 | -0.106 | 2.731 | -0.850 | 0.401 | 0.826 | -4.664 |
|  | P105-PWY | -0.270 | -1.854 | -0.845 | 0.404 | 0.826 | -4.665 |
|  | P341-PWY | 1.130 | -6.738 | 0.845 | 0.404 | 0.826 | -4.665 |
|  | PWY-5747 | -0.120 | 0.919 | -0.838 | 0.408 | 0.826 | -4.667 |
|  | PWY0-42 | -0.120 | 0.919 | -0.838 | 0.408 | 0.826 | -4.667 |
|  | PWY-7238 | -0.091 | 1.772 | -0.812 | 0.422 | 0.826 | -4.673 |
|  | PWY-7254 | -0.121 | 0.186 | -0.727 | 0.472 | 0.826 | -4.693 |
|  | PWY-6071 | 0.152 | -0.354 | 0.721 | 0.476 | 0.826 | -4.694 |
|  | PWY-5431 | 0.151 | -0.447 | 0.721 | 0.476 | 0.826 | -4.694 |
|  | SUCSYN-PWY | 0.992 | -3.654 | 0.692 | 0.493 | 0.826 | -4.700 |
|  | PENTOSE-P-PWY | -0.070 | 2.063 | -0.652 | 0.519 | 0.826 | -4.708 |
|  | PWY-7347 | 0.819 | -4.252 | 0.639 | 0.527 | 0.826 | -4.711 |
|  | GLYCOLYSIS-E-D | -0.086 | 1.415 | -0.637 | 0.528 | 0.826 | -4.711 |
|  | PWY-7118 | -0.076 | 1.674 | -0.636 | 0.529 | 0.826 | -4.712 |
|  | PWY-5913 | 0.101 | 1.282 | 0.624 | 0.537 | 0.826 | -4.714 |
|  | METH-ACETATE-PWY | -0.203 | -0.429 | -0.617 | 0.541 | 0.826 | -4.715 |
|  | PWY-6992 | -0.143 | -0.170 | -0.613 | 0.544 | 0.826 | -4.716 |
|  | CODH-PWY | -0.196 | -0.499 | -0.608 | 0.547 | 0.826 | -4.717 |
|  | PWY-5677 | -0.202 | -2.945 | -0.591 | 0.559 | 0.827 | -4.720 |
|  | PWY-5100 | -0.057 | 0.854 | -0.463 | 0.647 | 0.936 | -4.740 |
|  | PWY-6317 | -0.084 | 1.718 | -0.442 | 0.661 | 0.936 | -4.743 |
|  | PWY-3801 | 0.725 | -4.505 | 0.418 | 0.679 | 0.936 | -4.746 |
|  | PWY-6731 | -0.062 | 0.850 | -0.343 | 0.734 | 0.936 | -4.755 |
|  | PWY66-399 | -0.034 | 1.444 | -0.328 | 0.745 | 0.936 | -4.757 |
|  | P108-PWY | -0.095 | -1.375 | -0.290 | 0.773 | 0.936 | -4.760 |
|  | ANAEROFRUCAT-PWY | 0.029 | 1.409 | 0.290 | 0.773 | 0.936 | -4.760 |
|  | LACTOSECAT-PWY | -0.381 | -7.440 | -0.289 | 0.774 | 0.936 | -4.760 |
|  | PWY-6902 | -0.065 | 1.352 | -0.285 | 0.778 | 0.936 | -4.761 |
|  | PWY-6906 | -0.084 | -1.135 | -0.268 | 0.790 | 0.936 | -4.762 |
|  | ANAGLYCOLYSIS-PWY | 0.028 | 1.940 | 0.260 | 0.796 | 0.936 | -4.763 |
|  | METHANOGENESIS-PWY | -0.121 | -1.849 | -0.259 | 0.797 | 0.936 | -4.763 |
|  | PWY-5494 | -0.316 | -5.659 | -0.223 | 0.825 | 0.936 | -4.766 |
|  | PWY-7784 | -0.065 | -0.812 | -0.220 | 0.828 | 0.936 | -4.766 |
|  | PWY-8190 | -0.324 | -5.492 | -0.214 | 0.832 | 0.936 | -4.766 |
|  | PWY-622 | 0.019 | 1.762 | 0.210 | 0.835 | 0.936 | -4.766 |
|  | 3-HYDROXYPHENYLACETATE-DEGRADATION-PWY | 0.047 | -2.905 | 0.169 | 0.867 | 0.946 | -4.769 |
|  | PWY-7480 | 0.035 | -0.257 | 0.165 | 0.870 | 0.946 | -4.769 |
|  | PWY-7345 | 0.012 | 1.994 | 0.115 | 0.909 | 0.963 | -4.771 |
|  | PWY-5647 | -0.041 | -0.018 | -0.112 | 0.911 | 0.963 | -4.771 |
|  | PWY-5392 | 0.039 | -2.932 | 0.071 | 0.944 | 0.984 | -4.772 |
|  | PWY4LZ-257 | -0.038 | -4.740 | -0.027 | 0.979 | 0.984 | -4.773 |
|  | PWY-6588 | -0.009 | -2.485 | -0.022 | 0.983 | 0.984 | -4.773 |
|  | PWY-1882 | 0.024 | -4.244 | 0.021 | 0.984 | 0.984 | -4.773 |
| N vs RB | PWY-6071 | 0.677 | -0.354 | 3.201 | 0.003 | 0.083 | -1.658 |
|  | REDCITCYC | 0.679 | 1.132 | 3.194 | 0.003 | 0.083 | -1.672 |
|  | PWY0-321 | 0.435 | 0.367 | 3.052 | 0.004 | 0.083 | -1.978 |
|  | PWY-5431 | 0.636 | -0.447 | 3.042 | 0.004 | 0.083 | -2.000 |
|  | PWY-6435 | 0.410 | 1.008 | 2.864 | 0.007 | 0.083 | -2.370 |
|  | PWY-5742 | 0.569 | -0.852 | 2.852 | 0.007 | 0.083 | -2.395 |
|  | PWY-5913 | 0.458 | 1.282 | 2.823 | 0.008 | 0.083 | -2.454 |
|  | PWY-5430 | 0.507 | -0.670 | 2.564 | 0.015 | 0.137 | -2.965 |
|  | PWY-5130 | -0.288 | 2.141 | -1.952 | 0.059 | 0.485 | -4.041 |
|  | PWY-5677 | -0.652 | -2.945 | -1.902 | 0.066 | 0.485 | -4.120 |
|  | PWY-7347 | 2.237 | -4.252 | 1.745 | 0.090 | 0.580 | -4.355 |
|  | SUCSYN-PWY | 2.468 | -3.654 | 1.722 | 0.094 | 0.580 | -4.388 |
|  | PWY-5209 | 0.623 | -0.701 | 1.675 | 0.103 | 0.586 | -4.453 |
|  | P42-PWY | -0.207 | 2.557 | -1.588 | 0.121 | 0.598 | -4.573 |
|  | CALVIN-PWY | -0.185 | 2.501 | -1.578 | 0.124 | 0.598 | -4.586 |
|  | PWY-7254 | 0.249 | 0.186 | 1.495 | 0.144 | 0.598 | -4.694 |
|  | PWY-6906 | -0.457 | -1.135 | -1.463 | 0.153 | 0.598 | -4.735 |
|  | PWY-6641 | -0.347 | 1.226 | -1.434 | 0.161 | 0.598 | -4.770 |
|  | PWY-8187 | -0.259 | 1.586 | -1.433 | 0.161 | 0.598 | -4.771 |
|  | P461-PWY | -0.306 | 0.105 | -1.431 | 0.162 | 0.598 | -4.774 |
|  | P163-PWY | -0.343 | 0.064 | -1.355 | 0.184 | 0.604 | -4.865 |
|  | PWY-6969 | -0.163 | 2.578 | -1.336 | 0.190 | 0.604 | -4.886 |
|  | PWY-5384 | -0.151 | 2.350 | -1.316 | 0.197 | 0.604 | -4.909 |
|  | PWY-8178 | -0.162 | 2.652 | -1.309 | 0.199 | 0.604 | -4.917 |
|  | PWY-5392 | -0.700 | -2.932 | -1.279 | 0.209 | 0.604 | -4.950 |
|  | NONOXIPENT-PWY | -0.150 | 2.653 | -1.251 | 0.219 | 0.604 | -4.981 |
|  | PWY-6317 | -0.235 | 1.718 | -1.240 | 0.223 | 0.604 | -4.993 |
|  | GLUCONEO-PWY | -0.146 | 2.401 | -1.226 | 0.228 | 0.604 | -5.008 |
|  | PWY-3481 | 0.856 | -2.917 | 1.152 | 0.257 | 0.615 | -5.084 |
|  | PWY-7323 | -0.135 | 2.244 | -1.136 | 0.264 | 0.615 | -5.101 |
|  | PWY-7111 | -0.122 | 2.423 | -1.104 | 0.277 | 0.615 | -5.133 |
|  | PWY-5100 | -0.132 | 0.854 | -1.067 | 0.293 | 0.615 | -5.167 |
|  | 3-HYDROXYPHENYLACETATE-DEGRADATION-PWY | -0.292 | -2.905 | -1.057 | 0.298 | 0.615 | -5.177 |
|  | P23-PWY | -0.119 | 2.449 | -1.046 | 0.303 | 0.615 | -5.187 |
|  | PWY-1042 | -0.130 | 2.731 | -1.042 | 0.305 | 0.615 | -5.190 |
|  | CODH-PWY | -0.334 | -0.499 | -1.036 | 0.308 | 0.615 | -5.196 |
|  | METH-ACETATE-PWY | -0.334 | -0.429 | -1.013 | 0.318 | 0.615 | -5.217 |
|  | PWY-7238 | -0.113 | 1.772 | -1.003 | 0.323 | 0.615 | -5.225 |
|  | PWY-6628 | -0.109 | 2.330 | -1.000 | 0.324 | 0.615 | -5.228 |
|  | P108-PWY | -0.320 | -1.375 | -0.981 | 0.333 | 0.616 | -5.245 |
|  | PWY-622 | 0.088 | 1.762 | 0.964 | 0.342 | 0.617 | -5.259 |
|  | COMPLETE-ARO-PWY | -0.103 | 2.473 | -0.940 | 0.354 | 0.624 | -5.280 |
|  | PWY-5659 | -0.103 | 2.531 | -0.917 | 0.366 | 0.627 | -5.299 |
|  | GLYCOLYSIS | -0.102 | 2.427 | -0.903 | 0.373 | 0.627 | -5.310 |
|  | PWY-6731 | -0.155 | 0.850 | -0.849 | 0.402 | 0.639 | -5.352 |
|  | PWY-5484 | -0.092 | 2.360 | -0.845 | 0.404 | 0.639 | -5.355 |
|  | FERMENTATION-PWY | -0.094 | 1.977 | -0.832 | 0.411 | 0.639 | -5.365 |
|  | PWY-6588 | 0.354 | -2.485 | 0.826 | 0.415 | 0.639 | -5.369 |
|  | PWY-1882 | -0.871 | -4.244 | -0.741 | 0.464 | 0.701 | -5.429 |
|  | PWY4LZ-257 | -1.026 | -4.740 | -0.715 | 0.479 | 0.709 | -5.445 |
|  | LACTOSECAT-PWY | -0.808 | -7.440 | -0.613 | 0.544 | 0.789 | -5.506 |
|  | PWY-7118 | 0.069 | 1.674 | 0.570 | 0.572 | 0.814 | -5.529 |
|  | ANAGLYCOLYSIS-PWY | 0.053 | 1.940 | 0.483 | 0.632 | 0.864 | -5.571 |
|  | PWY-5747 | 0.067 | 0.919 | 0.469 | 0.642 | 0.864 | -5.577 |
|  | PWY0-42 | 0.067 | 0.919 | 0.469 | 0.642 | 0.864 | -5.577 |
|  | PWY-6902 | -0.102 | 1.352 | -0.450 | 0.656 | 0.864 | -5.584 |
|  | P341-PWY | -0.584 | -6.738 | -0.436 | 0.665 | 0.864 | -5.590 |
|  | PWY-7345 | 0.041 | 1.994 | 0.404 | 0.689 | 0.879 | -5.602 |
|  | GLYCOLYSIS-E-D | 0.045 | 1.415 | 0.338 | 0.737 | 0.925 | -5.624 |
|  | PWY-6992 | -0.070 | -0.170 | -0.301 | 0.765 | 0.944 | -5.635 |
|  | METHANOGENESIS-PWY | -0.128 | -1.849 | -0.274 | 0.786 | 0.945 | -5.642 |
|  | PWY-7784 | -0.070 | -0.812 | -0.237 | 0.814 | 0.945 | -5.651 |
|  | PENTOSE-P-PWY | 0.025 | 2.063 | 0.228 | 0.821 | 0.945 | -5.653 |
|  | PWY-5647 | -0.052 | -0.018 | -0.145 | 0.885 | 0.945 | -5.667 |
|  | ANAEROFRUCAT-PWY | 0.011 | 1.409 | 0.112 | 0.912 | 0.945 | -5.671 |
|  | PWY-5022 | -0.018 | 1.239 | -0.108 | 0.914 | 0.945 | -5.671 |
|  | PWY-7431 | 0.019 | 0.444 | 0.106 | 0.916 | 0.945 | -5.671 |
|  | PWY66-373 | 0.032 | -2.172 | 0.103 | 0.919 | 0.945 | -5.672 |
|  | PWY-3801 | -0.174 | -4.505 | -0.100 | 0.921 | 0.945 | -5.672 |
|  | PWY66-399 | 0.009 | 1.444 | 0.089 | 0.930 | 0.945 | -5.673 |
|  | PWY-5494 | -0.125 | -5.659 | -0.088 | 0.930 | 0.945 | -5.673 |
|  | PWY-7480 | 0.017 | -0.257 | 0.081 | 0.936 | 0.945 | -5.673 |
|  | PWY-8190 | -0.112 | -5.492 | -0.074 | 0.942 | 0.945 | -5.674 |
|  | P105-PWY | -0.022 | -1.854 | -0.070 | 0.945 | 0.945 | -5.674 |
| RC vs RB | PWY-6071 | 0.524 | -0.354 | 2.480 | 0.018 | 0.513 | -3.950 |
|  | PWY-5431 | 0.485 | -0.447 | 2.321 | 0.026 | 0.513 | -4.040 |
|  | REDCITCYC | 0.476 | 1.132 | 2.238 | 0.032 | 0.513 | -4.086 |
|  | PWY-7254 | 0.370 | 0.186 | 2.222 | 0.033 | 0.513 | -4.095 |
|  | PWY-5913 | 0.357 | 1.282 | 2.199 | 0.035 | 0.513 | -4.107 |
|  | PWY0-321 | 0.300 | 0.367 | 2.101 | 0.043 | 0.531 | -4.159 |
|  | PWY-5742 | 0.341 | -0.852 | 1.711 | 0.096 | 0.908 | -4.352 |
|  | PWY-6435 | 0.243 | 1.008 | 1.700 | 0.098 | 0.908 | -4.357 |
|  | PWY-5430 | 0.319 | -0.670 | 1.616 | 0.115 | 0.948 | -4.395 |
|  | PWY-5392 | -0.739 | -2.932 | -1.350 | 0.186 | 0.988 | -4.504 |
|  | PWY-5677 | -0.449 | -2.945 | -1.311 | 0.199 | 0.988 | -4.519 |
|  | PWY-5747 | 0.188 | 0.919 | 1.307 | 0.200 | 0.988 | -4.521 |
|  | PWY0-42 | 0.188 | 0.919 | 1.307 | 0.200 | 0.988 | -4.521 |
|  | P341-PWY | -1.713 | -6.738 | -1.281 | 0.209 | 0.988 | -4.530 |
|  | 3-HYDROXYPHENYLACETATE-DEGRADATION-PWY | -0.339 | -2.905 | -1.226 | 0.228 | 0.988 | -4.550 |
|  | PWY-7118 | 0.145 | 1.674 | 1.206 | 0.236 | 0.988 | -4.557 |
|  | PWY-6906 | -0.373 | -1.135 | -1.194 | 0.241 | 0.988 | -4.561 |
|  | PWY-7431 | 0.206 | 0.444 | 1.170 | 0.250 | 0.988 | -4.569 |
|  | PWY-7347 | 1.418 | -4.252 | 1.106 | 0.276 | 0.988 | -4.590 |
|  | SUCSYN-PWY | 1.476 | -3.654 | 1.030 | 0.310 | 0.988 | -4.614 |
|  | GLYCOLYSIS-E-D | 0.131 | 1.415 | 0.976 | 0.336 | 0.988 | -4.630 |
|  | PWY66-373 | -0.286 | -2.172 | -0.912 | 0.368 | 0.988 | -4.648 |
|  | PWY-5130 | -0.130 | 2.141 | -0.885 | 0.382 | 0.988 | -4.655 |
|  | PENTOSE-P-PWY | 0.095 | 2.063 | 0.880 | 0.385 | 0.988 | -4.656 |
|  | PWY-6588 | 0.364 | -2.485 | 0.847 | 0.403 | 0.988 | -4.664 |
|  | PWY-5022 | 0.136 | 1.239 | 0.835 | 0.409 | 0.988 | -4.667 |
|  | PWY-6317 | -0.151 | 1.718 | -0.798 | 0.430 | 0.988 | -4.677 |
|  | P105-PWY | 0.248 | -1.854 | 0.775 | 0.444 | 0.988 | -4.682 |
|  | PWY-1882 | -0.895 | -4.244 | -0.761 | 0.452 | 0.988 | -4.685 |
|  | PWY-622 | 0.069 | 1.762 | 0.754 | 0.456 | 0.988 | -4.687 |
|  | P108-PWY | -0.225 | -1.375 | -0.691 | 0.494 | 0.988 | -4.700 |
|  | PWY4LZ-257 | -0.988 | -4.740 | -0.689 | 0.496 | 0.988 | -4.701 |
|  | PWY-5100 | -0.075 | 0.854 | -0.604 | 0.550 | 0.988 | -4.717 |
|  | PWY-8187 | -0.096 | 1.586 | -0.531 | 0.599 | 0.988 | -4.730 |
|  | PWY-3801 | -0.898 | -4.505 | -0.518 | 0.608 | 0.988 | -4.732 |
|  | PWY-6731 | -0.092 | 0.850 | -0.506 | 0.616 | 0.988 | -4.734 |
|  | PWY-5209 | 0.176 | -0.701 | 0.473 | 0.639 | 0.988 | -4.739 |
|  | P42-PWY | -0.060 | 2.557 | -0.463 | 0.646 | 0.988 | -4.740 |
|  | FERMENTATION-PWY | 0.052 | 1.977 | 0.460 | 0.648 | 0.988 | -4.741 |
|  | P163-PWY | -0.112 | 0.064 | -0.442 | 0.661 | 0.988 | -4.743 |
|  | CODH-PWY | -0.138 | -0.499 | -0.427 | 0.672 | 0.988 | -4.745 |
|  | PWY66-399 | 0.044 | 1.444 | 0.416 | 0.680 | 0.988 | -4.747 |
|  | METH-ACETATE-PWY | -0.131 | -0.429 | -0.397 | 0.694 | 0.988 | -4.749 |
|  | CALVIN-PWY | -0.041 | 2.501 | -0.349 | 0.729 | 0.988 | -4.754 |
|  | LACTOSECAT-PWY | -0.427 | -7.440 | -0.324 | 0.748 | 0.988 | -4.757 |
|  | PWY-6992 | 0.073 | -0.170 | 0.312 | 0.757 | 0.988 | -4.758 |
|  | PWY-7345 | 0.029 | 1.994 | 0.289 | 0.774 | 0.988 | -4.760 |
|  | PWY-6969 | -0.035 | 2.578 | -0.285 | 0.777 | 0.988 | -4.761 |
|  | PWY-6641 | -0.065 | 1.226 | -0.270 | 0.788 | 0.988 | -4.762 |
|  | PWY-8178 | -0.033 | 2.652 | -0.268 | 0.791 | 0.988 | -4.762 |
|  | PWY-3481 | -0.174 | -2.917 | -0.234 | 0.816 | 0.988 | -4.765 |
|  | PWY-7323 | -0.027 | 2.244 | -0.229 | 0.820 | 0.988 | -4.765 |
|  | PWY-5384 | -0.026 | 2.350 | -0.226 | 0.822 | 0.988 | -4.765 |
|  | ANAGLYCOLYSIS-PWY | 0.024 | 1.940 | 0.223 | 0.825 | 0.988 | -4.766 |
|  | NONOXIPENT-PWY | -0.024 | 2.653 | -0.197 | 0.845 | 0.988 | -4.767 |
|  | PWY-1042 | -0.024 | 2.731 | -0.192 | 0.849 | 0.988 | -4.768 |
|  | PWY-7238 | -0.021 | 1.772 | -0.191 | 0.850 | 0.988 | -4.768 |
|  | ANAEROFRUCAT-PWY | -0.018 | 1.409 | -0.179 | 0.859 | 0.988 | -4.768 |
|  | PWY-6902 | -0.038 | 1.352 | -0.165 | 0.870 | 0.988 | -4.769 |
|  | PWY-7111 | 0.018 | 2.423 | 0.163 | 0.872 | 0.988 | -4.769 |
|  | P461-PWY | -0.033 | 0.105 | -0.156 | 0.877 | 0.988 | -4.769 |
|  | PWY-8190 | 0.212 | -5.492 | 0.140 | 0.890 | 0.988 | -4.770 |
|  | PWY-5494 | 0.190 | -5.659 | 0.134 | 0.894 | 0.988 | -4.770 |
|  | GLUCONEO-PWY | -0.016 | 2.401 | -0.130 | 0.897 | 0.988 | -4.771 |
|  | COMPLETE-ARO-PWY | 0.010 | 2.473 | 0.093 | 0.926 | 0.988 | -4.772 |
|  | P23-PWY | 0.010 | 2.449 | 0.084 | 0.933 | 0.988 | -4.772 |
|  | PWY-7480 | -0.018 | -0.257 | -0.084 | 0.934 | 0.988 | -4.772 |
|  | PWY-5659 | 0.006 | 2.531 | 0.057 | 0.955 | 0.988 | -4.773 |
|  | PWY-5484 | 0.004 | 2.360 | 0.041 | 0.968 | 0.988 | -4.773 |
|  | PWY-6628 | 0.004 | 2.330 | 0.035 | 0.972 | 0.988 | -4.773 |
|  | PWY-5647 | -0.012 | -0.018 | -0.033 | 0.974 | 0.988 | -4.773 |
|  | GLYCOLYSIS | 0.004 | 2.427 | 0.032 | 0.974 | 0.988 | -4.773 |
|  | PWY-7784 | -0.005 | -0.812 | -0.017 | 0.987 | 0.988 | -4.773 |
|  | METHANOGENESIS-PWY | -0.007 | -1.849 | -0.015 | 0.988 | 0.988 | -4.773 |

**Table S6** ANOVA table comparing microbial richness and Shannon diversity of microbial communities in vegetated low marsh and mid marsh, between N (Natural) and RC (Restored with creeks) salt marshes. Significant effects (p <0.05) are in bold.

|  |  | ChiSq | Df | p |
| --- | --- | --- | --- | --- |
| Microbial Richness | Restoration type | 3.569 | 1 | 0.059 |
|  | Elevation zone | 1.714 | 1 | 0.190 |
|  | Restoration type x Elevation zone | 0.002 | 1 | 0.966 |
| Shannon diversity | Restoration type | 0.194 | 1 | 0.660 |
|  | Elevation zone | 1.455 | 1 | 0.228 |
|  | Restoration type x Elevation zone | 0.450 | 1 | 0.503 |

**Table S7** Pairwise results of PERMANOVA comparing (a) microbial community structure and (b) microbial community composition between the restoration types (N: Natural, RC: Restored with creeks) and elevation zones (vegetated low marsh, vegetated mid marsh). Significant effects (p <0.05) are in bold.

(c) **Microbial community structure**

| Pairwise distinctions between restoration types | | | | | | |
| --- | --- | --- | --- | --- | --- | --- |
| Pairs | **Df** | **SS** | **F** | **R^2^** | **p** | **p-adj** |
| N – RC | 1 | 0.536 | 2.218 | 0.0460 | 0.022 | **0.022** |
| Pairwise distinctions between elevation zones | | | | | | |
| Pairs | **Df** | **SS** | **F** | **R^2^** | **p** | **p-adj** |
| Vegetated low marsh – Vegetated mid marsh | 1 | 1.687 | 7.790 | 0.145 | < 0.001 | **< 0.001** |

(d) **Microbial community composition**

| Pairwise distinctions between restoration types | | | | | | |
| --- | --- | --- | --- | --- | --- | --- |
| Pairs | **Df** | **SS** | **F** | **R^2^** | **p** | **p-adj** |
| N – RC | 1 | 0.536 | 2.218 | 0.0460 | 0.022 | **0.022** |
| Pairwise distinctions between elevation zones | | | | | | |
| Pairs | **Df** | **SS** | **F** | **R^2^** | **p** | **p-adj** |
| Vegetated low marsh – Vegetated mid marsh | 1 | 1.687 | 7.790 | 0.145 | < 0.001 | **< 0.001** |

**Table S8** ANOVA output from the distance-based redundancy analysis (dbRDA) plot, showing the sediment characteristics that explain the differences observed in the microbial community structure in the between the restoration types (N: Natural, RC: Restored with creeks) and elevation zones (vegetated low and mid marsh). Significant effects (p < 0.05) are in bold.

|  | Df | SS | F | p |
| --- | --- | --- | --- | --- |
| Nitrogen | 1 | 0.495 | 4.673 | **0.001** |
| Conductivity | 1 | 0.347 | 3.275 | **0.001** |
| pH | 1 | 0.184 | 1.737 | 0.045 |
| Sulphur | 1 | 0.263 | 2.484 | **0.004** |
| Silt-clay content | 1 | 0.266 | 2.510 | **0.004** |
| Organic matter | 1 | 0.366 | 3.458 | **0.001** |
| Residuals | 29 | 3.071 |  |  |

**Table S9** PERMANOVA and multivariate dispersion of the predicted carbon cycling pathway abundances across the restoration types (N: Natural, RC: Restored with creeks) and elevation zones (vegetated low marsh, vegetated mid marsh). Significant effects (p <0.05) are in bold.

|  | Df | SS | R^2^ | F | p |
| --- | --- | --- | --- | --- | --- |
| Restoration type x Elevation zone | 1 | 52.22 | 0.032 | 1.580 | 0.079 |
| Residual | 44 | 1454.46 | 0.896 |  |  |
| Permutation test for homogeneity of multivariate dispersion: Restoration type | | | | | |
|  | **Df** | **SS** | **MS** | **F** | **p** |
| Groups | 1 | 1.998 | 1.998 | 0.328 | 0.575 |
| Residuals | 46 | 280.418 | 6.096 |  |  |
| Permutation test for homogeneity of multivariate dispersion: Elevation zone | | | | | |
|  | **Df** | **SS** | **MS** | **F** | **p** |
| Groups | 1 | 0.309 | 0.309 | 0.049 | 0.816 |
| Residuals | 46 | 289.662 | 6.297 |  |  |
